# Towards Managing and Controlling Aflatoxin Producers Within *Aspergillus* Species in Infested Rice Grains Collected from Local Markets in Kenya

**DOI:** 10.3390/toxins11090544

**Published:** 2019-09-19

**Authors:** Youmma Douksouna, Joel Masanga, Andrew Nyerere, Steven Runo, Zachée Ambang

**Affiliations:** 1 Institute for Basic Sciences Technology and Innovation, Pan African University, P.O. Box 62000–00200 Nairobi, Kenya; 2 Department of Biochemistry, Microbiology and Biotechnology, Kenyatta University, P.O. Box 43844–00100 Nairobi, Kenya; 3 Department of Medical Microbiology, Jomo Kenyatta University of Agriculture and Technology, P.O. Box 62000–00200 Nairobi, Kenya; 4 Department of Plant Biology and Biotechnology, University of Yaounde1, P.O. Box 812 Yaoundé, Cameroon

**Keywords:** rice grains, *Aspergillus* species, prevalence, ITS, nor-1, ver-1, aflatoxigenic

## Abstract

Rice grains can be attacked by a range of pathogens, including *Aspergillus* species, which can cause the accumulation of aflatoxins and represent a serious threat to the consumers. Aflatoxins are secondary metabolites synthesized by *Aspergillus* species and naturally occur in various foodstuffs. In this study, we sought to analyze the prevalence of aflatoxin-producing *Aspergillus* spp. in rice grains currently sold in Kenyan local markets. We analyzed a total of 98 samples randomly collected and primarily analyzed to observe moisture content and fungal growth. We then isolated *Aspergillus* species, characterized them morphologically and using the Internal transcribed spacer (ITS) primers. Finally, we screened them for aflatoxin-producing isolates targeting Norsolorinic Acid (*nor-1*) and *Versicolorin* (*ver-1*) specific genes involved in aflatoxin biosynthesis. We observed that all tested samples were contaminated. The highest prevalence of *Aspergillus* species and aflatoxigenic fungal species, had values of 66% and 36.4% for *nor-1* and *ver-1*, respectively. In total, 66% of all isolates were confirmed to be aflatoxin producers. The occurrence of high contamination levels of *Aspergillus* species points to the possibility of production of aflatoxins in rice grains. This work provides a baseline for future studies on the occurrence of mycotoxigenic fungal species in rice grains being sold in local markets and strategies to control these aflatoxigenic strains at pre- and post-harvest levels.

## 1. Introduction

Rice is a cereal consumed by a great part of the human population throughout the world. This consumption is in many forms, including products such as white rice, parboiled rice, meal rice, and rice bran [1]. Generally, dried rice grains stored under inappropriate conditions, such as damp/misty places, insect infestation, and favorable environmental conditions of temperature and humidity, are likely to provide a viable substrate for the growth of fungi [2,3]. Mycotoxin contamination could further occur during the processing of rice grains [4]. Additionally, a delayed drying process and excess moisture (above 13%) [5] can promote the growth of fungi. Numerous species in *Aspergillus* section *Flavi* are common in plants and their processed derivatives, with some producing diverse mycotoxins, such as aflatoxins, 3-nitropropionic acid, tenuazonic acid, and cyclopiazonic acid [6]. The production of aflatoxins is associated with spore production by species of *Aspergillus* [7]. Four variants of aflatoxins, including aflatoxin B_1_ (AFB_1_), B_2_ (AFB_2_), G_1_ (AFG_1_), and G_2_ (AFG_2_), are produced by *Aspergillus* spp. and these have been described to have carcinogenic effects, as well as mutagenic and teratogenic capabilities. Furthermore, these compounds are known to result in immunosuppression [8]. Consequently, the International Agency for Research on Cancer has classified these compounds as group I carcinogens and mutagens with immunosuppressive properties and they can inhibit several metabolic systems [9]. About 5.2 million cancer deaths occur each year, 55% of which occur in developing countries [10]. The removal of aflatoxins (AFs) is difficult due to their stability and thermal resistance in dried products [11]. In fact, AFs are resistant to food processing and thus may remain throughout the food chain [12]. Therefore, AFS are potential threats to human health, either by the direct consumption of contaminated food products or by carry over aflatoxins and their metabolites in milk and meat [13]. World health authorities warn that low doses with long-term dietary exposure to aflatoxins represent a major risk of hepatocellular carcinoma [14]. The current study therefore aimed at isolating and characterizing *Aspergillus* strain contaminants from rice collected from markets in Kenya, with the aim of generating knowledge on the possible consumption of and exposure of the local population to these carcinogens.

## 2. Results and Discussion

### 2.1. Results

#### 2.1.1. Moisture Content

**Figure 1:**
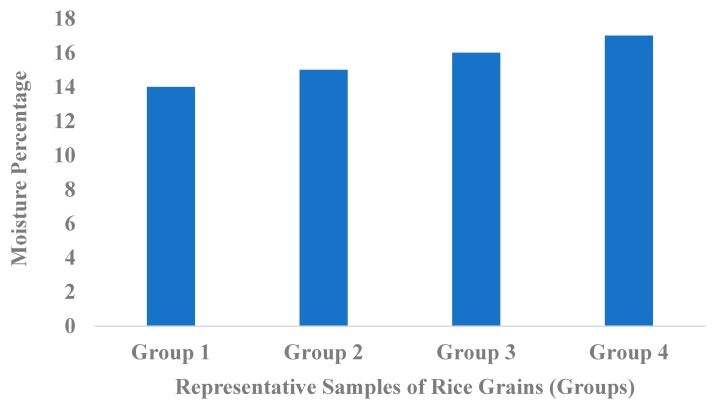
Clustering of samples and the basis of their moisture content.

In the present study, mycoflora was isolated from rice grains to evaluate the contamination level in rice grains sold in the market. Samples collected were previously examined for moisture content and the prevalence of *Aspergillus* species. Environmental conditions, the critical ones being temperature and a high moisture content, influence fungal growth in foodstuffs. All samples were initially analyzed for moisture content to check for the relationship correlation (Y = 4.211 × X − 54.39) between the moisture level in rice grains and fungal growth. Four groups of samples were clustered according to their percentages: 14–14.5%, 15–15.5%, 15.6–16.5% and 16.6–17.9%, respectively, for group 1, group 2, group 3 and group 4, as shown in Figure 1. 

Samples with high moisture contents for a length of time offer the best microclimate for the growth of mycotoxigenic fungi and mycotoxins production. A positive correlation between moisture content and the number of isolates was observed in this study (R^2^ = 0.2926). With the increase of moisture content, the number of isolates was increased, indicating a possibly higher level of fungal growth and contamination of rice grains in group 3 and 4 samples. 

#### 2.1.2. Isolation of *Aspergillus* Species 

The fungal isolation results revealed the presence of *Aspergillus* in all samples. From these observations, all inoculated plates except for the negative control indicated that the rice grains were colonized by fungi.

#### 2.1.3. Morphological Analysis

**Figure 2:**
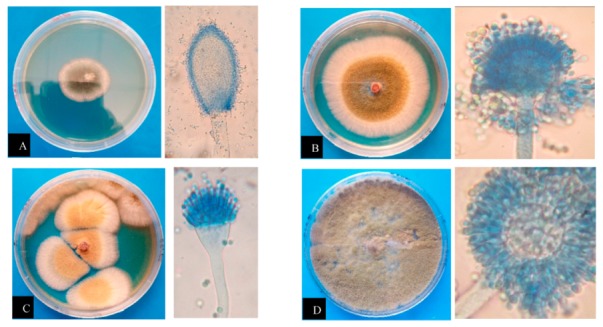
Left to right: 7-day-old colonies on potato dextrose agar (PDA) and malt extract agar (MEA); top to bottom: (**A**) *Aspergillus clavatus*; (**B**) *Aspergillus flavus*; (**C**) *Aspergillus fumigatus*; (**D**) *Aspergillus parasiticus*.

All the samples were positive for *Aspergillus*, with a total of eight (8) fungal strains, including, *Aspergillus clavatus*, *Aspergillus flavus*, *Aspergillus fumigatus*, *Aspergillus nomius*, *Aspergillus oryzae*, *Aspergillus parasiticus*, *Aspergillus versicolor*, and *Aspergillus* spp., being observed. These strains were purified based on their distinct morphology on malt extract agar (MEA) and potato dextrose agar (PDA) plates and differentiated based on their macroscopic and microscopic characteristics. Macroscopic identification was based on colony and reverse color, diameter, exudates, and texture. On the basis of the macroscopic study, isolates could not be differentiated. Further microscopic study involved the arrangement, color, diameter, shape, size, wall characters, cellular contents, conidial heads, conidiophore, sterigmata, conidia, and conidial arrangements, which were observed with a microscope [15]. The macroscopy and microscopy characteristics of fungal isolates are presented in Figure 2.

**Figure 3:**
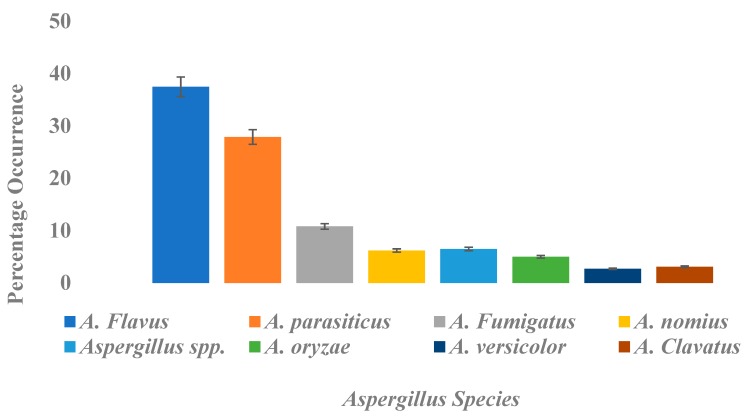
Occurrence of different *Aspergillus* species isolated from rice grains presented as a percentage.

Morphological analysis revealed that *Aspergillus flavus* (37.5%) was the most represented strain, followed by *Aspergillus parasiticus* (27.9%), *Aspergillus fumigatus* (10.8%), *Aspergillus nomius* (6.2%), *Aspergillus oryzae* (5%), *Aspergillus clavatus* (3.1%), *Aspergillus versicolor* (2.7%), and *Aspergillus* spp. (6.5%) (Figure 3). The ability of fungi to change their morphology at different stages of growth and reproduction renders phenotypic characterization inefficient. Therefore, these isolates were subjected to molecular analysis through polymerase chain reaction (PCR) amplification of their Internally Transcribed Spacer (ITS) regions and a two gene profile of *Aspergillus* species involved in the aflatoxin biosynthetic pathway *Norsolorinic Acid* (*NOR*): *aflD* (*nor-1*), and *Versicolorin*: *aflM* (*ver-1*) fragments of aflatoxigenic fungal genomic DNA.

#### 2.1.4. Molecular Characterization

**Figure 4:**
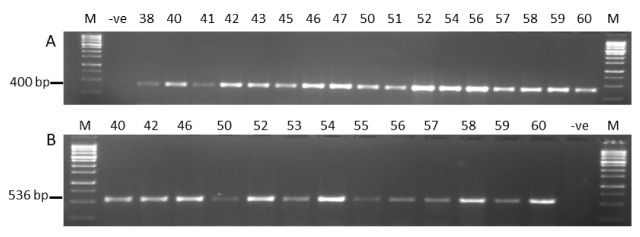
Polymerase chain reaction (PCR) amplicons of (**A**) the 400 bp product of *aflD* and (**B**) the 536 bp product of *aflM* markers. M: molecular weight marker 1 kb (Solis Biodyne, Tartu, Estonia); -ve: negative control. Numbers are the code of samples Figure S4.

Genomic DNA was isolated from 74 samples of *Aspergillus* strains for molecular analysis. The quantity of extracted DNA from all the samples was between 369 µg/mL and 1998 µg/mL, which is an excellent quantity range. Genomic DNA was successfully amplified, revealing the presence of three genes across various samples. The expected size of each primer pair produced a single DNA fragment of 598 bp for ITS (Figure S1), and 400 and 536 bp for *nor-1* and *ver-1*, respectively Figures S2 and S3. The results of the amplified products are shown in Figure 4. From a total of 74 phenotypically characterized *Aspergillus* species isolates, all samples showed positive PCR results for the ITS primer pair. Although ITS1-ITS4 is the most commonly used primer pair for the detection of *Aspergillus* species [16], it does not have a sufficient discriminative power to differentiate between aflatoxigenic and non-aflatoxigenic (atoxigenic) species. Therefore, primer pairs for the specific genes profile involved in the aflatoxin biosynthetic pathways *nor-1* and *ver-1* were amplified. Altogether, 74 samples were screened, in which 49 samples were found to be positive for *nor-1* (27 for *Aspergillus flavus*, 8 for *Aspergillus nomius*, and 14 *Aspergillus parasiticus*), and 27 were found to be positive for *ver-1* (14 for *Aspergillus flavus,* 5 for *Aspergillus nomius*, and 8 *Aspergillus parasiticus*). No amplification was observed for other strains, including *Aspergillus fumigatus*, *Aspergillus oryzae*, *Aspergillus clavatus*, and *Aspergillus versicolor*.

### 2.2. Discussion

Contamination of foodstuffs by mycotoxin-producing fungi is on the rise in the world, with most cereals and grains being affected. These fungi lead to the deterioration of food products through the production of harmful toxins, such as aflatoxins. Key among these is *Aspergillus* spp., which synthesize aflatoxins. Moisture content is one of the determining factors for the growth of *Aspergillus* species [17]. In the current study, rice grains from local markets showed a high moisture content (14% to 17.9%), which is above the standard levels of 12%–13% for rice grains reported [18]. These results are in line with those reported by Hina [19], who attributed the same to processing, warm and humid weather, and poor handling of grains. 

In order to understand if rice grains from Kenyan local markets are contaminated by mycotoxin-producing fungi, we analyzed the prevalence of *Aspergillus* species. The results showed the occurrence of fungal species in all the samples, with *Aspergillus flavus* being the most predominant, followed by *Aspergillus parasiticus*, as compared to other strains. This can be attributed to the ability of *Aspergillus flavus* to grow on a variety of substrates across different environmental conditions, as well as its capability to produce numerous spores that remain viable, even under extreme conditions. These findings are in agreement with reports by several other studies who revealed a high prevalence of *Aspergillus* species in the contamination of rice grains from local markets [19,20,21]. In our case, this contamination might be due to the exposure of rice grains to fungal colonization during processing, transportation, and storage. Poor handling conditions, including improper packaging and poor ventilation, as well as increased humidity, in the ware houses could further be exacerbating the growth of fungi in stored grains, as described previously [22]. The high prevalence of *Aspergillus* species compared to other fungal isolates in the current study could be a pointer towards the contamination of these grains by aflatoxins [23]. Due to the toxic and carcinogenic properties of aflatoxins, there is an urgent need to develop a sensitive, rapid, and specific technique for the identification of aflatoxin produced from food samples. In our study, the PCR reaction was targeted against ITS1-ITS4 for the detection of *Aspergillus* species, and the *nor-1* and *ver-1* genes profile targeted DNA for screening aflatoxigenic strains from *Aspergillus* isolated. The primers ITS1 and ITS4 previously successfully amplified all the ITS region isolates selected using conventional PCR. In gel electrophoresis of the PCR product, an amplicon corresponding to 598 bp in size was only seen in a positive sample, which clearly indicated that the primers (ITS1-ITS4) were specific for *Aspergillus* species. This result is in concordance with that recorded by many investigators [16,24], showing that ITS1-ITS4 is the most commonly used primer pair for the detection of *Aspergillus* species. 

A look at the process of aflatoxin production in these fungi shows that the biosynthetic pathway comprises many enzymatic steps that lead to the end product. *Aspergillus* species within the genus show a variation in aflatoxin production, with some species not able to synthesize aflatoxins, and these are termed atoxigenic [25]. Apart from a functional analysis of aflatoxin production in *Aspergillus*, several studies have reported the use of PCR technology as a rapid and sensitive method for the detection and diagnosis of aflatoxin production [26], as well as the detection of aflatoxigenic strains from non-aflatoxigenic counterparts [27,28].

In the current study, we used PCR for the detection of potential aflatoxigenic *Aspergillus* strains infecting rice grains currently sold at Kenyan markets. We targeted *nor-1* (*aflD*) and *ver-1* (*aflM*); key structural genes in the aflatoxin biosynthetic pathway that play a key role in the synthesis of the toxin. Our results showed that a high number of *Aspergillus* strains (66%) were positive for *nor-1*, whilst 36% of the isolates were positive for *ver-1*. This indicated that these isolates are potential aflatoxin producers based on the role played by the two genes during aflatoxin biosynthesis. On the other hand, isolates that showed negative PCR results for these genes indicated that these isolates could be non-aflatoxigenic. It is possible that this non-aflatoxigenicity is due to mutations or deletions of these genes that inhibit the role they play during toxin biosynthesis [29]. This is in line with Houshyarfard et al. [30], who reported that the production of aflatoxin is bound to several factors, including the presence of certain genes and the nature of these genes. This means that there should not be major deletions or insertions within the gene regions or regions flanking the gene. Otherwise, deletions of several portions of the aflatoxin biosynthesis gene cluster have been reported to be the main cause for the lack of aflatoxin production. Keeping in view the above results, it is obvious that the contamination level of mycotoxigenic fungi in rice grains sold at the markets is very high. Our results are in accordance with other reports demonstrating the importance of PCR assay techniques for detecting the aflatoxigenic potential of *Aspergillus* strains [20,21,31,32,33,34].

## 3. Conclusions 

On the basis of the achieved results, we conclude that the prevalence of *Aspergillus* species in rice grains sold at the markets is high, and indicates the possible high level of aflatoxigenic strains in rice grains under study. In this study, 66% of isolates were used to confirm aflatoxin production genes considered as indicators of aflatoxin production. This is indicative of exposure of population to aflatoxins and could lead to possible health problems. Amplification of aflatoxin and specific genes proved a rapid and accurate detection system to differentiate the aflatoxigenic and non-aflatoxigenic isolates. Further studies on the sequencing and quantification of aflatoxins in rice grains and their relationship with the level of contaminating *Aspergillus* species are in progress.

## 4. Materials and Methods

### 4.1. Samples 

Samples comprising local and imported rice grains were collected randomly (1 kg) from local retail markets and millers in Mwea and Thika (Kenya) and labelled appropriately. A total of 98 samples (local rice produced in Mwea and imported rice originating from Biriyani, India, Pakistan, and Thailand) were taken according to the alternative sampling plan for the official control of mycotoxins in food [35]. Representative samples were then put in sealed bags and transported to the Molecular Biology and Biotechnology Laboratory in Pan African University institute for Basic Sciences, Technology and Innovation (PAUSTI).

### 4.2. Moisture Content Analysis

To better understand the factors that may have led to aflatoxin contamination of the samples, each sample of rice grains collected was analyzed for its moisture content using a Grainer meter (Model Japonica, Miami, FL, USA).

### 4.3. Isolation and Enumeration of Fungal Species

Isolation of microflora from the grains was conducted following the method of Ulster [36]. Samples of rice grains were analyzed by the direct plating method described by Pitt et al. [37]. For each sample, 20 pieces (rice grains suspected to be contaminated by fungi) were placed aseptically on a layer of moistened filter paper in the Petri dishes. The Petri dishes were incubated in an upright position at 25 °C for 5–7 days in darkness and ventilated for 12 h on the 3rd day. After incubation, the plates were examined, and the number of contaminated particles was counted and reported as a percentage. Fungi growing on different seeds were isolated from emerging colonies on modified rose Bengal chloramphenicol agar (*MRBA*) [38]. Pure cultures were carried out for subsequent studies using two (2) culture media: malt extract agar (MEA) and potato dextrose agar (PDA), according to Pitt et al., [39] and Varga et al. [40], for seven days.

### 4.4. Phenotypic Characterization

Phenotypic characterization was done on the basis of the mycelium growth pattern, color, and properties of fruiting bodies of the fungi. The growth pattern of fungal colonies, colony size (diameter) and color (reverse and coarse), exudates, and colony margins were the macroscopic features used in identification. The size of the head, vesicle shape, phailides, matulae, conidiophores, and conidia (conidial diameter, wall, shape, surface, and conidia attachment with conidiophore) were employed for microscopic identification. These features were finally compared with the synoptic keys for identification of the isolated fungi [26].

The percentage occurrence of each species isolated was also calculated by the following formula:

$$\%\;\mathrm{Occurrence}\;\mathrm{of}\;\mathrm{species}=(\frac{\mathrm{No}.\;\mathrm{of}\;\mathrm{isolates}\;\mathrm{of}\;a\;\mathrm{species}}{\mathrm{Total}\;\mathrm{no}.\;\mathrm{of}\;\mathrm{isolates}\;\times \;100})$$

### 4.5. Molecular Characterization of the Isolates

The genomic DNA was extracted from seven-day-old fungi cultures grown on MEA and PDA media and the fungal mass from the pure cultures was scraped-out from the plates. A total of 50–100 mg of fungal mycelium was scraped and placed in a 2 mL tube and vortexed vigorously for 30 min with glass beads to crush the mycelial wall and to release the DNA. A volume of 500 µL of Lysis Buffer (100 mM Tris-HCl pH 8, 1 mM EDTA, 100 mM NaCl, 10 mM B-mercaptoethanol, and 1% Sodium dodecyl sulfate), 5 µL of RNase A, and 1 µL of Proteinase K were added. Tubes were then incubated at 65 °C for 45 min in buffer. 

Thereafter, 270 µL of Sodium/Potassium acetate (3 M) was added and samples were well-mixed and centrifuged at 13,000 rpm for 10 min. After centrifugation supernatant (700 µL) was transferred to a fresh tube, and an equal volume of chloroform: isoamylalcohol (24:1) was added and mixed well, samples were stood on a bench for 5 min, followed by centrifugation at 13,000 rpm for 10 min. The supernatant (700 µL) was transferred into new tubes, and 80 µL of Sodium/Potassium acetate (3M) and 587 µL of ice cold isopropanol were added and then mixed well by inverting the tubes and then incubating them at −20 °C overnight. After that, samples were centrifuged at 13,000 rpm for 30 min, and the supernatant was discarded carefully. The DNA pellets were washed with 1 mL of 70% ethanol and centrifuged at 13,000 rpm for 10 min. The DNA pellets were air dried and dissolved in 50 µL of Tris-EDTA (TE) buffer and stored at −20 °C until use.

Separation of the isolated genomic DNA was done using agarose gel electrophoresis, followed by Sybr Green visualization using Lambda (λ) as a DNA size marker. The concentration and purity of genomic DNA were measured using a Nano-drop Spectrophotometer (Model PCR Max Lambda, Thermo Fisher Scientific, Waltham, MA, USA), and the purity of all extracted DNA was measured by taking their absorbance at 260 nm and 280 nm.

### 4.6. Diagnostic PCR Using AspergillusUniversal Primers

We used *Aspergillus* universal primer pairs to amplify the ITS1 and ITS4 region of different isolated *Aspergillus* strains for characterization and primer sequences already available in the literature [32] to amplify two different genes involved in the Aflatoxin biosynthetic pathway *Norsolorinic Acid* (*NOR*): *aflD* (*nor-1*), and *Versicolorin*: *aflM* (*ver-1*) fragments of aflatoxigenic fungal genomic DNA. The sequences of primers are listed in Table 1.

PCR analysis was performed in a 20 µL reaction mixture comprising 1 µL of genomic DNA, 4 µL of premix Taq buffer (Solis Biodyne, Tartu, Estonia), and 0.5 µL of each forward and reverse primer. The final volume was made up to 20 µL with nuclease free water. Amplification was performed in a Proflex PCR System (Model 4483636, Thermo Fisher Scientific, Waltham, MA, USA) with the following conditions (Table 2).

The amplified PCR products were resolved by gel electrophoresis in a 1.5% agarose (Sigma, St. Loaus, MO, USA) gel stained in 10 µL of Trugel Fluorescent Dye. The DNA bands resolved on agarose gel were visualized in the UV Documentation system with a camera (Model UV Doc. HDS UITEC Cambridge, UK) transilluminator. The sizes of the amplicon were estimated by comparing them with a commercial 1 kb DNA ladder on agarose gel.

## Figures and Tables

**Table 1:** Oligonucleotide primer sets used for the study.

Set	Primer Name	Sequences (5′–3′)	Length of PCR Product (bp)
1	ITS1	F-TCCGTAGGTGAACCTGCGG	598
	ITS4	R-TCCTCCGCTTATTGATATGC	
2	*aflD*	F-ACCGCTACGCCGGCACTCTCGGCAC	400
		R-GTTGGCCGCCAGCTTCGACACTCCG	
3	*aflM*	F-GCCGCAGGCCGCGGAGAAAGTGGT	536
		R-GGGGATATACTCCCGCGACACAGCC	

Abbreviations: F, forward; R, reverse.

**Table 2:** Polymerase chain reaction (PCR) parameters of the oligonucleotide primer sets used for the study.

Set	PCR Fragment	Initial Denaturation		Denaturation		Annealing		Elongation		Final Elongation		Number of Cycles
1	ITS	94 °C	5 min	95 °C	60 s	52 °C	60 s	72 °C	60 s	72 °C	10 min	35
2	aflD	94 °C	5 min	94 °C	60 s	64 °C	60 s	72 °C	60 s	72 °C	10 min	33
3	aflM	95 °C	5 min	95 °C	60 s	65 °C	60 s	72 °C	2 min	72 °C	10 min	33

## Supplementary Materials

The following are available online at https://www.mdpi.com/2072-6651/11/9/544/s1, Figure S1: Gel images of ITS1 and ITS4, Figure S2: Gel images of *aflD* aflD (400bp product), Figure S3: Gel images of *aﬂM* (536bp product), Figure S4: Run of some positive samples for publication, Document S1: Screening of aflD and aflM.
