# Supplementary material for: Towards Managing and Controlling Aflatoxin Producers Within Aspergillus Species in Infested Rice Grains Collected from Local Markets in Kenya
Source: Toxins (Basel). 2019 Sep 19;11(9):544. doi: 10.3390/toxins11090544 (PMC6784266; doi:10.3390/toxins11090544)
Supplement: Supplementary file 1 [file toxins-11-00544-s001.zip › toxins-562875 SI.docx]

Supplementary Materials: Towards Managing and Controlling Aflatoxin Producers Within *Aspergillus* Species in Infested Rice Grains Collected from Local Markets in Kenya

Youmma Douksouna, Joel Masanga, Andrew Nyerere, Steven Runo and Zachée Ambang


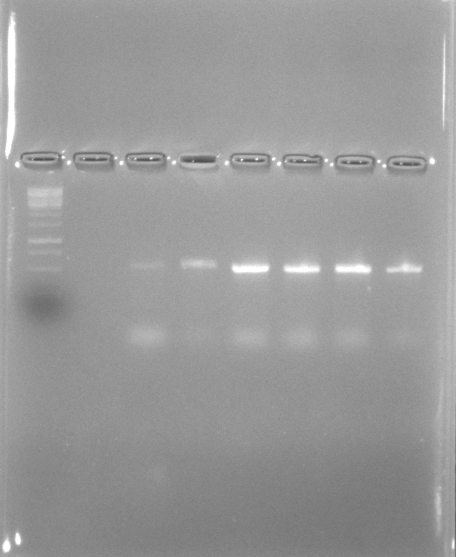


598

Figure S1. Gel images of ITS1 and ITS4

M -ve 1 2 3 4 5 6 7 8 9 10 11 12 13 14 15 16 17 M


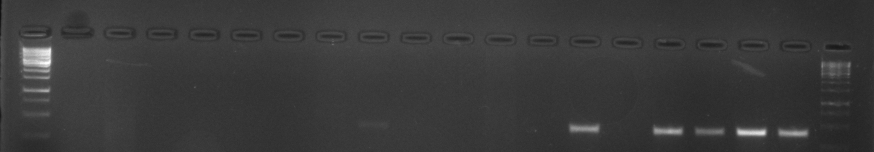


**Figure 2.** Gel images of *aflD* (400bp product). M: molecular weight marker 1kb (Solis Biodyne), -ve: negative control. Numbers are the code of Samples.

M -ve 18 19 20 21 22 23 24 25 26 27 28 29 30 31 31 33 34 M


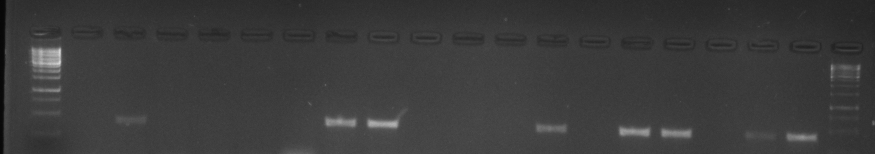


M -ve 35 36 37 38 39 40 41 42 43 44 45 46 47 48 49 50 51 M


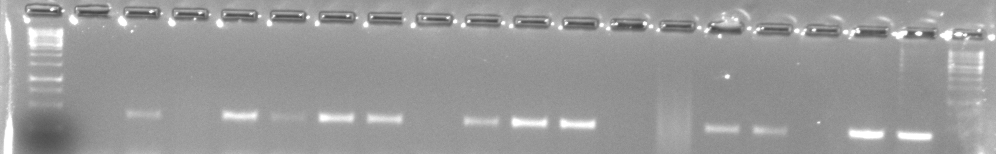


M 52 53 54 55 56 57 58 59 60 61 62 63 64 65 66 67 68 -ve M


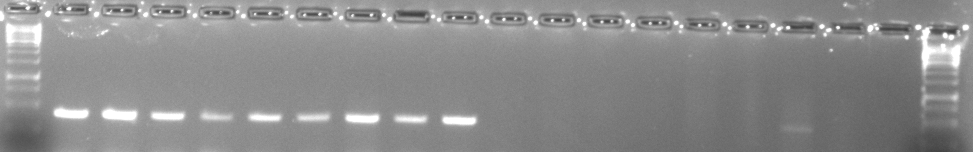


M -ve 69 70 71 72 73 74 M


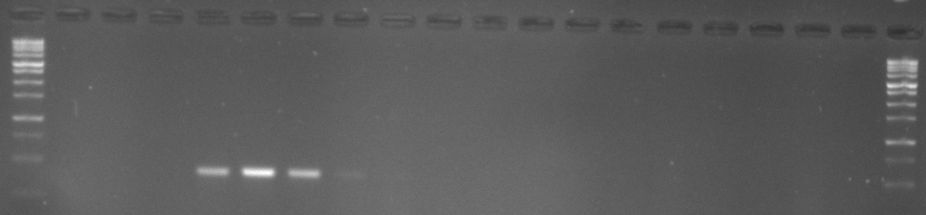


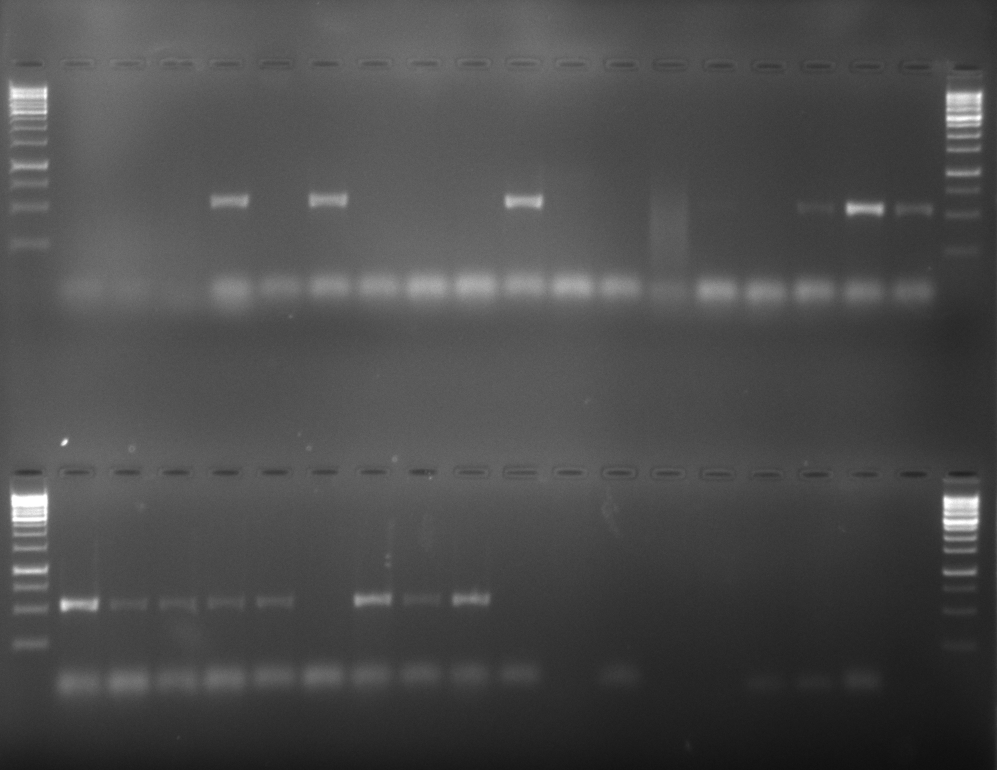


M 55 56 57 58 59 60 61 62 63 64 65 67 68 69 70 71 72 73 M M

M -ve 38 39 40 41 42 43 44 45 46 47 48 49 50 51 52 53 54 M M

**Figure S3.** Gel images of *aﬂM* (536bp product). M: molecular weight marker 1kb (Solis Biodyne), -ve: negative control. Numbers are the code of Samples.


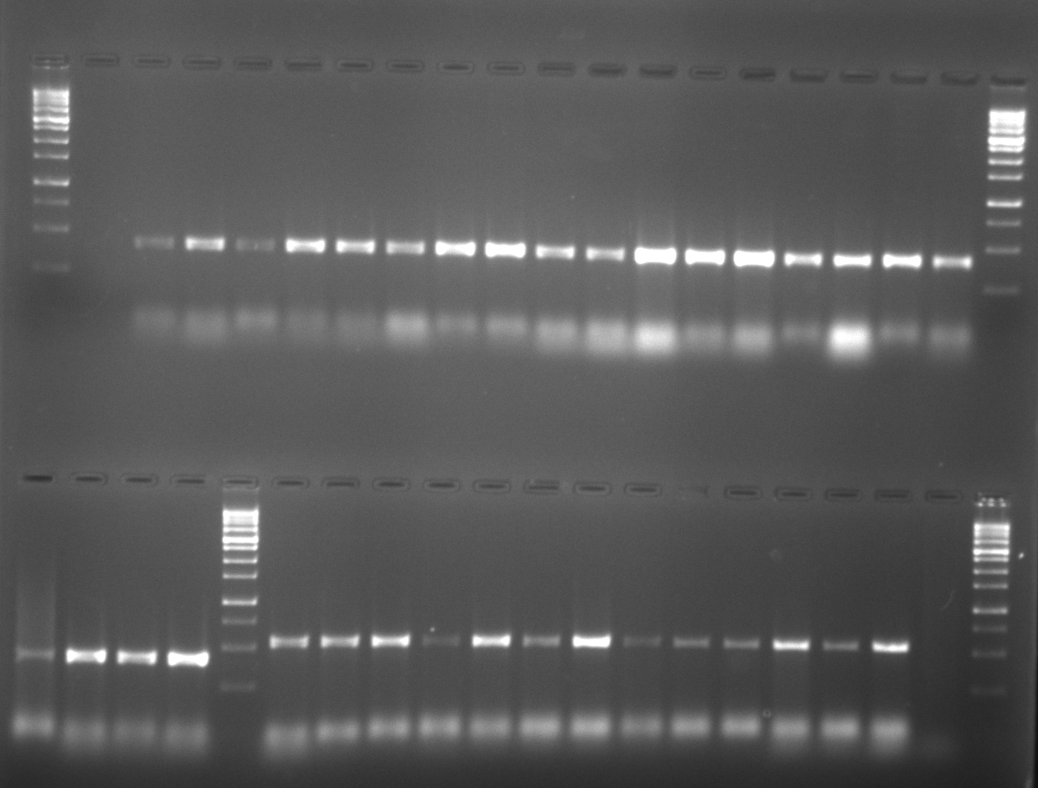


*aﬂM* (536bp product)

*aflD* (400bp product)

*aflD* (400bp product)

**Figure S4.** Run of some positive samples for publication.
